# Clinical characterization of NTCP deficiency in paediatric patients : A case‐control study based on *SLC10A1* genotyping analysis

**DOI:** 10.1111/liv.15031

**Published:** 2021-08-25

**Authors:** Li‐Jing Deng, Wen‐Xian Ouyang, Rui Liu, Mei Deng, Jian‐Wu Qiu, Muhammad‐Rauf Yaqub, Muhammad‐Atif Raza, Wei‐Xia Lin, Li Guo, Hua Li, Feng‐Ping Chen, Ying Ouyang, Yu‐Ge Huang, Yue‐Jun Huang, Xiao‐Ling Long, Xiao‐Ling Huang, Shuang‐Jie Li, Yuan‐Zong Song

**Affiliations:** ^1^ Department of Paediatrics The First Affiliated Hospital of Jinan University Guangzhou China; ^2^ Department of Hepatopathy Hunan Children’s Hospital Changsha China; ^3^ Department of Laboratory Science The First Affiliated Hospital of Jinan University Guangzhou China; ^4^ Department of Paediatrics Sun Yat‐Sen Memorial Hospital Sun Yat‐Sen University Guangzhou China; ^5^ Department of Paediatrics The Affiliated Hospital of Guangdong Medical University Zhanjiang China; ^6^ Department of Paediatrics The Second Affiliated Hospital of Shantou University Medical College Shantou China; ^7^ Department of Paediatrics Bo‐Ai Hospital of Zhongshan Zhongshan China; ^8^ Dongguan Maternal and Child Health Care Hospital Dongguan China

**Keywords:** cholestatic jaundice, hypercholanemia, indirect hyperbilirubinemia, *SLC10A1* gene

## Abstract

Na^+^‐taurocholate cotransporting polypeptide deficiency (NTCPD) is a newly described disorder arising from biallelic mutations of the *SLC10A1* gene. As a result of a lack of compelling evidence from case‐control studies, its genotypic and phenotypic features remain open for in‐depth investigation. This study aimed to explore the genotypic and clinical phenotypic characteristics of paediatric patients with NTCPD. The *SLC10A1* genotypes of all NTCPD patients were confirmed by screening for the prevalent variant c.800C>T and Sanger sequencing when necessary. The clinical presentations and laboratory changes were collected, reviewed and analysed, and then qualitatively and quantitatively compared with the relevant controls. A total of 113 paediatric NTCPD patients were diagnosed while c.374dupG and c.682_683delCT were detected as two novel pathogenic mutations. Hypercholanemia was observed in 99.12% of the patients. Indirect hyperbilirubinemia in affected neonates exhibited higher positive rates in comparison to controls. Moreover, transient cholestatic jaundice, elevated liver enzymes and 25‐hydroxyvitamin D (Vit D) deficiency during early infancy were more commonly observed in patients than in controls. All NTCPD patients exhibited favourable clinical outcomes as a result of symptomatic and supportive treatment. The findings enriched the *SLC10A1* mutation spectrum and provided comprehensive insights into the phenotypic characteristics of NTCPD. NTCPD should be considered and *SLC10A1* gene should be analysed in patients with above age‐dependent clinical features. Furthermore, over investigation and intervention should be avoided in the management of NTCPD patients.

## 1 INTRODUCTION

Na^+^‐taurocholate cotransporting polypeptide (NTCP) is a protein encoded by the solute carrier family 10 member 1 (*SLC10A1*) gene, which is exclusively expressed in the basolateral membrane of the hepatocyte, functioning to uptake conjugated bile acids from plasma into hepatocyte to maintain the enterohepatic circulation of bile acids.^1^, ^2^ The human NTCP gene was cloned in 1994. Since then, the NTCP function has been studied extensively,^3^, ^4^ and in particular, mice‐based findings have substantially advanced the understanding of the pathophysiology of NTCP deficiency (NTCPD).^5^, ^6^, ^7^ However, for years, human NTCPD remained a mystery in terms of the genotypic and phenotypic presentations.

This situation changed in 2015 when Vaz et al described the first NTCPD patient in the world.^8^ Following this, increasing numbers of patients with this disorder have been described, who were clinically characterized by nearly negative manifestations other than hypercholanemia, while some pathogenic/likely‐pathogenic *SLC10A1* variants have been identified.^9^, ^10^, ^11^, ^12^, ^13^, ^14^, ^15^, ^16^ These molecular and clinical findings provided very important contribution to the understanding of human NTCPD as an autosomal recessive disorder. However, previous NTCPD patients were largely reported as sporadic cases or case series, and compelling molecular and clinical evidence is still in need to provide comprehensive insights into the genotypic and phenotypic characteristics of this new inborn error of bile acid metabolism. This study established a large cohort of paediatric patients with NTCPD, and the *SLC10A1* variant spectrum and clinical presentations were profiled.

## 2 MATERIALS AND METHODS

### 2.1 Subjects and ethics

The research subjects in this study included 113 paediatric patients with NTCPD (Table S1) who were definitely diagnosed from 173 paediatric patients with hypercholanemia and their family members (Figure S1), and the initial presentations of the research subjects were summarized in Table S2. Once the diagnosis of NTCPD was made through *SLC10A1* analysis, all clinical data (including the symptoms, signs, history, biochemistry data and clinical outcomes) of the paediatric patients before the diagnosis and on follow‐up were collected for subsequent analysis. Hence, the same patient may have multiple data for every biochemistry index including total bile acids (TBA).

Moreover, 52 healthy children were enrolled as non‐NTCPD controls for the comparison of relevant biochemical indices. As the common controls for the positive rate survey of neonatal indirect hyperbilirubinemia and cholestatic jaundice in early infancy, a total of 367 neonates (Figure S2), who were born from October 19 to November 30, 2019, in the Department of Obstetrics, the First Affiliated Hospital, Jinan University, were observed and followed until January 2020.

The allele frequencies of the identified novel *SLC10A1* mutations in the local population were investigated by way of Sanger sequencing, using the peripheral blood samples from 60 healthy volunteers who underwent health examination in our hospital.

This study adheres to the World Medical Association Declaration of Helsinki (WMADH2008), which was adopted by the 59th WMA General Assembly, Seoul, in October 2008, and has been approved by the Medical Ethical Committee, the First Affiliated Hospital, Jinan University (No. KY‐2019‐052). Written informed consents were also obtained from the research subjects or their guardians prior to the study.

### 2.2 Clinical evaluation

The history of all patients was primarily collected by interviewing the children's caregivers. All patients were physically examined by at least one of the authors. The laboratory indices were mainly analysed in the Department of Laboratory Science, the First Affiliated Hospital, Jinan University, and some clinical and biochemical data were collected from the medical records provided by parents of the patients at their referral to our hospital. The biochemical data from other hospitals were standardized when necessary.

### 2.3 Biochemistry assay

All the 113 research subjects in this study (Table S1) underwent biochemistry analysis because of the major complaints summarized in Table S2. Fasting peripheral blood samples were collected, and a total of 27 biochemistry indices, including serum alanine transaminase (ALT), aspartate transaminase (AST), gamma‐glutamyl transpeptidase (GGT), alkaline phosphatase (ALP), adenosine deaminase (ADA), choline esterase (CHE), total protein (TP), albumin (ALB), globulin (GLB), total bilirubin (TBIL), direct bilirubin (DBIL), TBA, cholyglycine (CG), total cholesterol (TCHOL), triglyceride (TG), high‐density lipoprotein (HDL), low‐density lipoprotein (LDL), apolipoprotein A (APOA), apolipoprotein B (APOB), apolipoprotein E (APOE), superoxide dismutase (SOD), ceruloplasmin (CER), fibronectin (FN), retinol‐binding protein (RBP) and zinc, were analysed using relevant commercial kits on a HITACHI 7600 automatic biochemical analyzer (Japan). Of note, TBA was tested by the assay Kit via Enzyme Cycle Method, while CG was tested using a commercially available kit by latex immunoturbidimetric assay. TBIL and DBIL were tested via the vanadate oxidation method using relevant assay kits and indirect bilirubin (IBIL) was calculated by subtracting DBIL from TBIL. Serum 25‐hydroxyvitamin D (Vit D) levels were analysed using a DIASORIN LIAISON automatic chemiluminescent immune analyzer (USA). The ages and numbers of testing varied from person to person depending on when and how many times they were followed up.

### 2.4 Diagnostic criteria

In this study, hypercholanemia denoted serum TBA levels beyond 40 μmol/L in neonates and 10 μmol/L after the neonatal period.

Neonatal indirect hyperbilirubinemia was in accordance with one of the following standards: (1) Jaundice appeared within 24 hours after birth. (2) The serum TBIL has reached the light therapy intervention standard in the corresponding age and risk factors,^17^ or increased by >85 μmol/L per day, or >0.85 μmol/L per hour. (3) Jaundice lasted for >2 weeks in full‐term and >4 weeks in premature infants. Any patients with cholestatic jaundice or with hemolytic diseases such as glucose‐6‐phosphate dehydrogenase (G6PD) deficiency, polycythemia, thalassemia, or ABO incompatibility were excluded.

Cholestatic jaundice was defined as the proportion of DBIL to TBIL >20% when TBIL >85 µmol/L, or DBIL >17.1 µmol/L when TBIL ≤85 µmol/L.^18^

In addition, the diagnosis of Vit D deficiency was made when the serum Vit D level was below 10 ng/mL.

### 2.5 Molecular analysis

Peripheral blood of 2 mL was collected from each subject, and the genomic DNA was extracted using a DNA extraction kit (Simgen, China) according to the manufacturer's instructions. The PCR‐RFLP analysis of the prevalent *SLC10A1* variant c.800C>T(p. Ser267Phe) was conducted as reported previously.^9^

In cases with single or no mutated *SLC10A1* allele detected, Sanger sequencing was performed to explore the hidden causative variant. The primers, thermocycling conditions, and product sequencing process were as previously described.^10^ Pathogenicity prediction was according to the Standards and Guidelines developed by American College of Medical Genetics and Genomics (ACMG).^19^ Allele frequency information was also obtained from 1000 Genomes project (1KGP), Exome Sequencing Project (ESP), Exome Aggregation Consortium (ExAC), The Human Gene Mutation Database (HGMD) and all relevant literature in PubMed, as previously described.^16^

### 2.6 Statistical analysis

Data were expressed as mean ± SD, median (range), or n (%) as appropriate. Spaghetti plots with trend lines were used to present the biochemical indices varying with time when necessary. All statistics were performed with R software (version 3.6.2, R Foundation for Statistical Computing). The Shapiro‐Wilk test was used for normal distribution test. Comparisons of multiple rates or component ratios were performed using the Pearson's Chi‐squared test, and when the minimum theoretical frequency is <1, or n < 40, using the Fisher exact test. A Kruskal‐Wallis test was used for comparing multiple groups, and the Wilcoxon rank‐sum test or *t* test was applied for comparing the two independent groups as appropriate. Spearman's rank correlation analyses between the TBA and bilirubin (TBIL, DBIL and IBIL) levels before phototherapy in neonatal period were performed respectively. *P* < .05 was considered a statistically significant criterion.

## 3 RESULTS

### 3.1 ***SLC10A1*** **genotyping results**

In this study, a total of 113 paediatric patients with NTCPD from 109 unrelated families were definitely diagnosed by *SLC10A1* analysis (Table S1). A total of five *SLC10A1* variants, that is, c.800C>T(p. Ser267Phe), c.263T>C (p. Ile88Thr), c.595A>C(p. Ser199Arg), and two novel frameshift mutations c.374dupG(p. Cys125TrpfsTer23) and c.682_683delCT (p. Leu228AspfsTer49), were detected in this large cohort, with c.800C>T(p. Ser267Phe) as a predominant variant accounting for 94.50% (206/218) of all mutated alleles. The two novel mutations had not been included in any PubMed literature, and both were negative in the databases of 1KGP, ESP, ExAC and HGMD, as well as in the 60 healthy volunteers in this study. According to the ACMG criteria, the *SLC10A1* variants detected in this study were classified as pathogenic or likely pathogenic (Table S3).

### 3.2 Hypercholanemia

**FIGURE 1:**
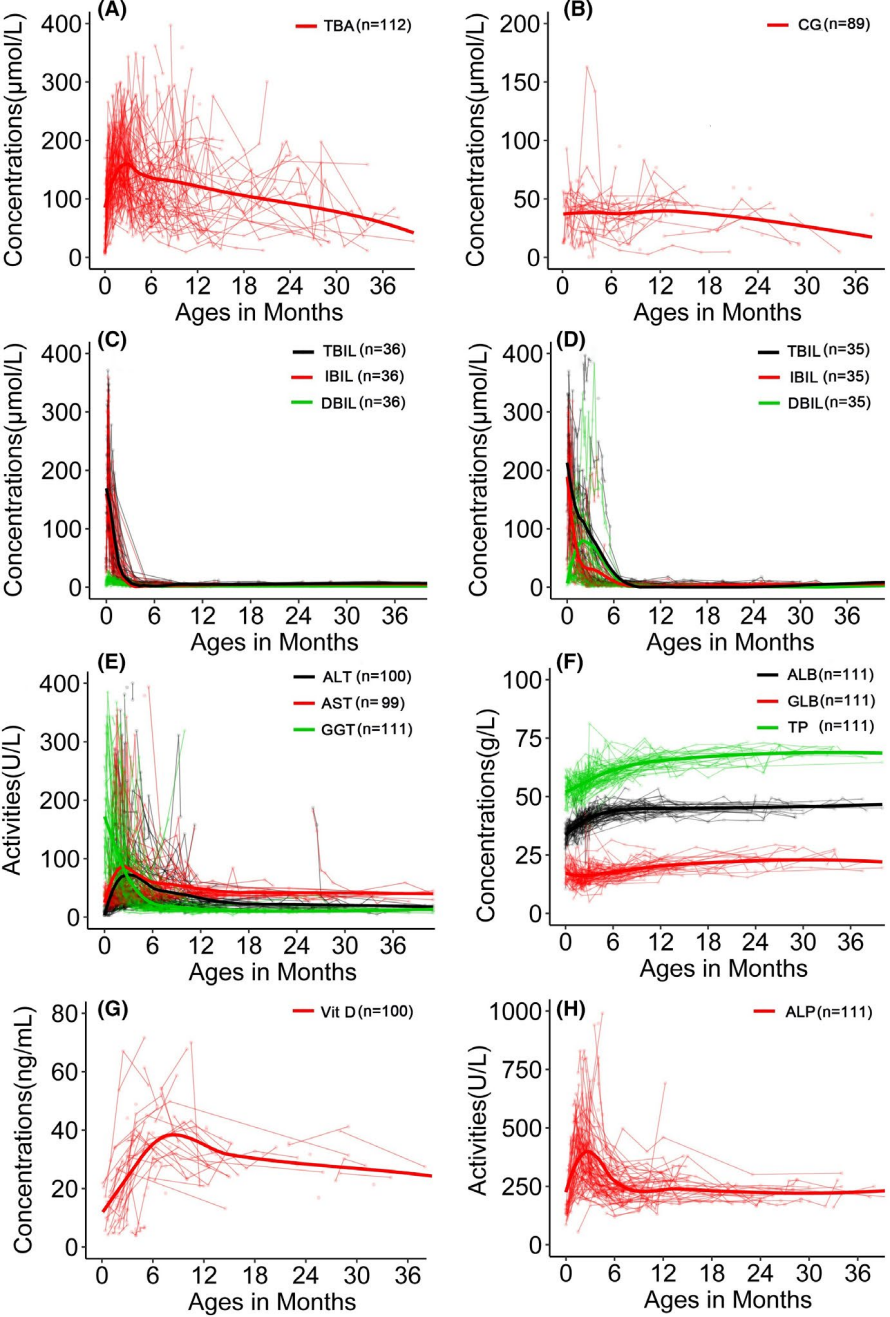
Biochemistry changes over time in the paediatric NTCPD patients. This figure described the longitudinal changes of the relevant biochemistry indices (with n provided on the upper right corner of every figure) collected from paediatric NTCPD patients with laboratory data. Figures A and B indicated the curves of TBA and CG over time respectively. Figures C and D depicted the bilirubin changes in paediatric patients with neonatal indirect hyperbilirubinemia and transient cholestatic jaundice in early infancy respectively. Figures E–H indicated the changes of ALT, AST, GGT, TP, ALB, GLB, Vit D and ALP over time respectively. All biochemistry indices in this figure were abbreviated as in the Biochemistry assay section of the MATERIALS AND METHODS

Among the research subjects with TBA results, hypercholanemia was found in nearly all paediatric patients, and the positive rate (99.12%, 112/113) in patients was significantly higher than 6.38% (3/47) in healthy controls (Table 1). Paediatric patients displayed higher TBA levels than healthy children (Table S4). The peak level occurred at 3‐4 months of age and the TBA levels thereafter tended downward over time but kept intractably raised within 36 months of age (Table 2; Figure 1A). Similar results were observed when analysing the serum CG levels among the same two groups (Table S4; Figure 1B).

**TABLE 1:** Clinical characteristics of NTCP deficiency in children

Clinical characteristics	Paediatric patients	Healthy children	Χ^2^	*P*
Hypercholanemia	99.12% (112/113)	6.38% (3/47)	136.65	<.001
Neonatal indirect hyperbilirubinemia	92.31% (36/39)	35.03% (103/294)	44.12	<.001
Cholestatic hepatitis in early infancy				
↑ DBIL	34.31% (35/102)	0.36% (1/274)	95.07	<.001
↑ ALT	63.33% (57/90)	18.75%(3/16)	9.25	.002
↑ AST	65.91%(58/88)	0% (0/16)	21.25	.001
↑ GGT	80.81% (80/99)	53.33%(8/15)	—	.041*
Vit D deficiency within 6 months of age	88.89% (8/9)	0% (0/16)	—	<.001*

* Fisher exact test was used.

**TABLE 2:** Comparison of the serum biochemical indices among the paediatric groups

Indices	Reference ranges	Paediatric patients (0‐28 d)	Paediatric patients (29 d‐6 m)	Paediatric patients (>6 m)	Healthy children (29 d‐6 m)	Healthy children (>6 m)	Χ^2^	*P* *
Number	—	39	102	75	20	32	—	—
Ages^a^	—	10.9 ± 8.3, n = 175	2.8 ± 1.4^▲^, n = 416	11.5 (6.1, 70)^■^, n = 274	2 (1, 6)^▲^, n = 16	48 (7, 180)^★^, n = 31	541.78	<.001
TBA^b^	0‐10 μmol/L	102.5 ± 56.0^▲^, n = 175	142 (28, 492.8)^■^, n = 416	108.9 (2.9, 737.8)^▲^, n = 274	7.8 ± 2.9^★^, n = 16	3.6 ± 2.0^◆^, n = 31	198.87	<.001
CG	0.4‐2.78 mg/L	84.8 (26.4, 201.7)^▲^, n = 29	87.1 (0.5, 353)^▲^, n = 104	76.4 ± 41.4^▲^, n = 101	3.4 (1.75, 7.72)^■^, n = 13	2.1 (0.23, 3.9)^■^, n = 25	94.98	<.001
TBIL	5.1‐23 μmol/L	156.8 ± 96.4^▲^, n = 164	21.1 (2.1, 540.8)^■^, n = 367	6.6 (1.8, 25.2)^★^, n = 237	12.9 (5.9, 28.2)^■^, n = 16	7.8 (2.5, 18.7)^◆★^, n = 31	404.79	<.001
IBIL	1.7‐17 μmol/L	135.7 ± 93.9^▲^, n = 146	10.5 (0.5, 223.3)^■^, n = 356	4.4 (0.5, 14.3)^★^, n = 227	8.5 (3.9, 22.7)^■^, n = 16	5.7 (0.6, 15)^◆★^, n = 31	380.96	<.001
DBIL	0.6‐6.8 μmol/L	13.6 (1.3, 193.4)^▲^, n = 223	6.5 (0, 394.1)^■^, n = 369	2 (0.1, 9.2)^★^, n = 246	4.0 (1.3, 8.8)^■^, n = 16	2 (0.8, 6.3)^★^, n = 31	374.73	<.001
ALT	5‐40 U/L	17 (3, 207)^▲^, n = 151	40 (10, 874)^■^, n = 344	23.5 (5, 1161)^★^,n = 242	28.6 ± 8.2^■★^, n = 16	17.4 ± 5.6^▲^, n = 31	193.06	<.001
AST	5‐50 U/L	37 (12, 232)^▲^, n = 131	49 (17, 1295)^■^, n = 334	40 (23, 482)^★^, n = 240	42 (22, 49)^▲■★^, n = 16	32 (15, 51)^▲★^, n = 31	91.48	<.001
GGT	10‐60 U/L	147 (18, 753)^▲^, n = 118	55 (6, 530)^■^, n = 336	12 (3, 65)^★^, n = 238	63 (13, 290)^■^, n = 15	10 (6, 19)^★^, n = 31	485.29	<.001
Vit D	>10 ng/mL	15.1 ± 7.0^▲^, n = 19	24.9 (4, 83.9)^■^, n = 101	31.9 (13.2, 70)^★^, n = 92	35 (21.1, 50.2)^■★^, n = 17	33.2 ± 11.2^■^, n = 19	47.61	.001

Note

Significant differences (*P* < .05) were represented by the distinct superscript patterns (▲,■,★,◆) on the right of the relevant values.

Abbreviations: D, day; M, month.

^a^ The ages when the biochemistry analysis was performed for TBA.

^b^ 40 µM as the upper limit for TBA in neonates.

* indicated the statistical differences among the five groups.

### 3.3 Neonatal indirect hyperbilirubinemia

Generally, the TBIL, DBIL and IBIL levels were elevated in paediatric NTCPD patients (Table S4). As shown in Table 2, when making age‐specific comparisons in the paediatric patients, the affected neonates exhibited highest indirect bilirubinemia levels. On Spearman's rank correlation analyses, TBA was found to be positively correlated with the bilirubin levels before phototherapy in the neonatal patients with NTCPD, as shown in Figure S3. Furthermore, qualitatively, neonatal indirect hyperbilirubinemia was revealed in 36 of the 39 paediatric NTCPD patients with neonatal data, with the positive rate of 92.31% (36/39) being significantly higher than the 35.03% (103/294) in neonatal controls (χ^2^ = 44.12, *P* < .001; Table 1). In response to phototherapy, the neonatal indirect hyperbilirubinemia in the 36 paediatric patients all recovered by 2.5 months after birth (Figure 1C).

### 3.4 Cholestatic hepatitis in early infancy

The serum DBIL, ALT, AST and GGT levels in paediatric NTCPD patients were significantly higher than in the healthy children (Table S4). Further comparison of the biochemistry data among the children grouped by age revealed that ALT and AST demonstrated peak levels when aged 29 days to six months (Table 2).

Qualitatively, transiently elevated DBIL, ALT, AST and GGT were observed in 34.31% (35/102), 63.33% (57/90), 65.91% (58/88) and 80.81% (80/99) of the paediatric NTCPD patients within six months of age, while in age‐matched common controls, their positive rates were just 0.36% (1/274, χ^2^ = 95.07, *P* < .001), 18.75% (3/16, χ^2^ = 9.25, *P* = .002), 0% (0/16, χ^2^ = 21.25, *P* < .001) and 53.33% (8/15, *P* = .041) respectively (Table 1).

**FIGURE 2:**
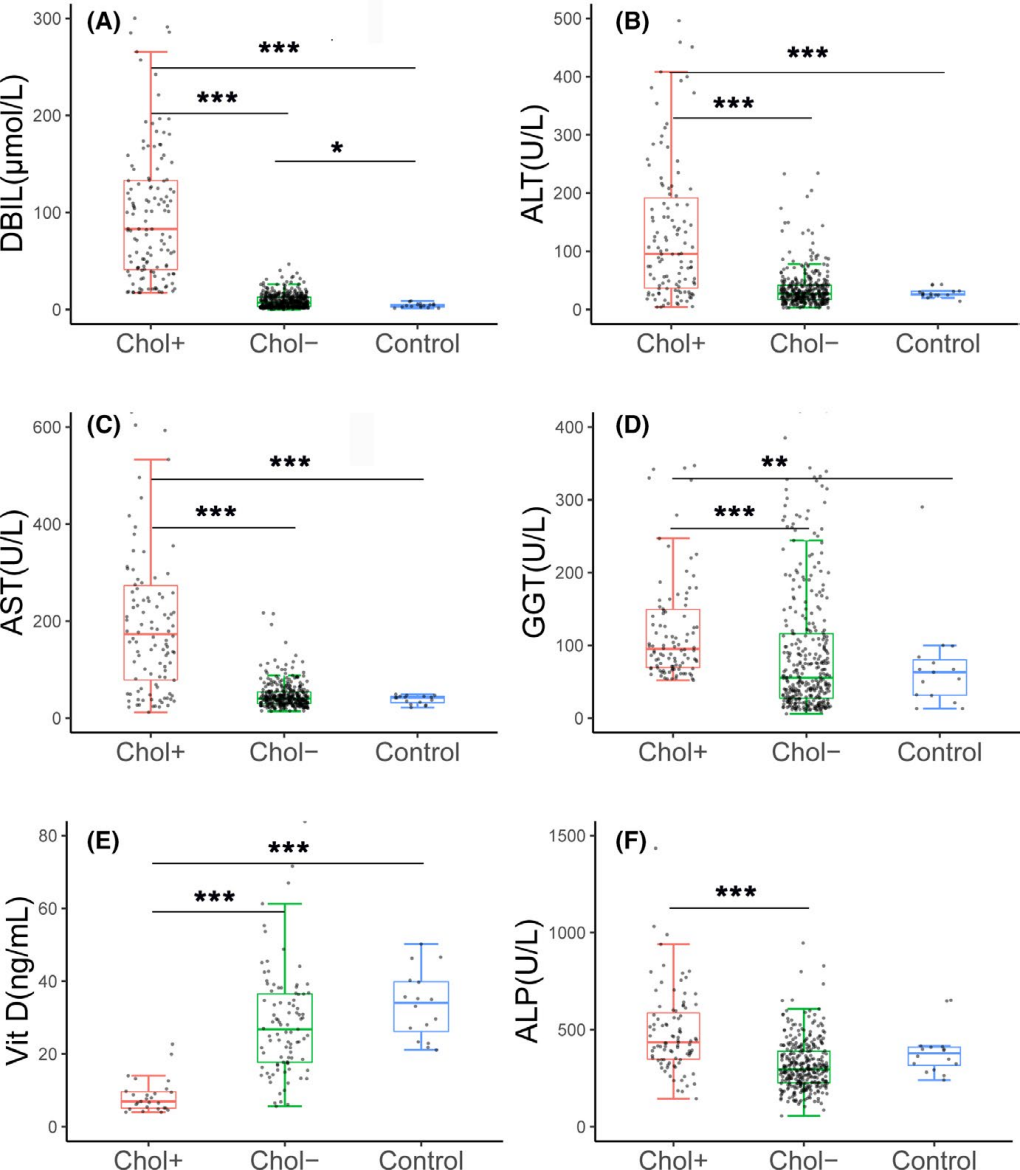
Comparison of the biochemical indices among different groups of infants within 6 months of age. (A–F) illustrated the comparison of the DBIL, ALT, AST, GGT, Vit D and ALP levels among the three groups. In this figure, “Chol +” = NTCPD patients with cholestasis, “Chol ‐” = NTCPD patients without cholestasis, while ***, ** and *P < .001, 0.001 ≤ *P* < .01 and 0.01 ≤ *P* < .05 respectively. All biochemistry indices were abbreviated as in the Biochemistry assay section of the MATERIALS AND METHODS

The elevated DBIL levels recovered before six months of life in the 35 paediatric NTCPD patients with cholestatic jaundice (Figure 1D), while the raised levels of ALT, AST both reached their peaks at 2‐4 months after birth while resolved within 6‐12 months of age (Figure 1E). Quantitatively, the cholestatic paediatric patients during early infancy also displayed DBIL, ALT, AST and GGT levels higher than age‐matched healthy controls, age‐matched (age < 6m) non‐cholestatic paediatric patients (Figure 2A‐D).

### 3.5 Vit D deficiency in NTCPD infants with cholestasis

When compared with other paediatric NTCPD groups, neonatal patients showed significantly decreased Vit D levels (Table 2; Figure 1G). Qualitatively, NTCPD patients with cholestasis by six months of age exhibited higher positive rate of Vit D deficiency when compared with the age‐matched NTCPD patients without cholestasis [88.89% (8/9) vs 6.32% (6/95), *P* < .001] and healthy infants [88.89% (8/9) vs 0% (0/16), *P *< .001] (Table 1). Similarly, ALP elevation was more commonly observed in NTCPD patients with cholestasis by six months of age than in age‐matched NTCPD patients without cholestasis[14.29% (4/28) vs 2.15%(2/93), *P* < .05]. Quantitatively, Vit D levels in NTCPD patients with cholestasis by six months of age were significantly lower, while ALP was higher, than the age‐matched patients without cholestasis (Figure 2E,F).

### 3.6 Other laboratory changes

No significant differences were observed between the paediatric patients and healthy children groups in terms of the serum levels of ADA, TG, TCHOL, HDL, LDL, APOB, APOE, FN, RBP, CHE, CER and Zinc (Table S4), whereas the serum levels of TP, ALB, GLB, APOA and SOD displayed statistically significant differences between the two groups (Table S4). However, since APOA and SOD both displayed means or medians within their reference ranges, the above differences were not clinically significant. Although TP, ALB and GLB levels were lower in paediatric patients than in healthy children (Table S4), they all rose with age and reached normal by 10‐12 months of age (Figure 1F), indicating a physiological maturation process.

### 3.7 Management and outcomes

No specific therapy was given other than symptomatic and supportive treatment including phototherapy for neonatal indirect hyperbilirubinemia as well as oral Vit D supplementation in patients with Vit D deficiency. The clinical and laboratory abnormalities other than hypercholanemia gradually disappeared or recovered in all paediatric patients, and of note, Vit D deficiency and ALP elevation both resolved gradually by the age six months (Figure 1G‐H) at the same rate as disappearance of cholestatic jaundice. However, for exploration of the cholestatic etiology, next‐generation sequencing (NGS) analysis was performed in eight affected infants while surgical operations were performed in five infant patients (Table S5).

## 4 DISCUSSION

The c.800C>T variant accounted for 94.50% (206/218) of all mutated *SLC10A1* alleles in this cohort of NTCPD patients. Since this variant reportedly impaired the entry of hepatitis B virus (HBV) into the hepatocyte,^4^ its prevalence may arise as a result of positive selection during a long evolution process.^20^ The novel mutations c.374dupG and c.682_683delCT were detected in trans with the pathogenic variant c.800C>T in the NTCPD patients, and theoretically resulted in formation of premature termination codons and hence production of the truncated NTCP proteins p. Cys125TrpfsTer23 and p. Leu228AspfsTer49 respectively. Moreover, they were both negative in publicly available databases and in the 60 healthy volunteers in this study. According to the ACMG guidelines and standards,^19^ they were diagnosed as pathogenic *SLC10A1* variants serving as genetic biomarkers for NTCPD patients (Table S3). This study established the hitherto largest cohort of NTCPD patients through *SLC10A1* genetic analysis, and the two novel variants expanded the *SLC10A1* mutation spectrum.

Hypercholanemia was the most common phenotypic feature for NTCPD in this study. In human adult volunteers, myrcludex B increased TBA exposure 19.2‐fold without signs of cholestasis, and the rise in conjugated bile acids was up to 123‐fold (taurocholic acid),^21^ and the profound hypercholanemia in normal adults induced by chemical inhibition of NTCP with myrcludex B suggested any contribution from any other transporter for conjugated bile acids unimportant. However, in this study, the downward tendency of hypercholanemia with increasing age (Figure 1A) as well as the occasionally normal TBA levels (Figure 1A; Table 2) both strongly indicated the existence of other auxiliary transport systems in humans to compensate for the impaired NTCP function. Currently, organic anion transporter polypeptides (OATP) 1B1/1B3 were considered as the main molecular replacement mechanism, which were localized to the basolateral membrane of human hepatocytes and mediated the Na^+^‐independent uptake of bilirubin, bile salts and other organic anions.^5^, ^22^, ^23^ In mice, the OATP1 expression at birth was just 15% of adult levels,^24^ and this may be an explanation for hypercholanemia to exhibit declining tendency with growing ages in the NTCPD patients, although the maturation mechanisms may differ among different species. In addition, there may be compensatory hepatic uptake of bile salts by way of the heterodimer organic solute transporters α/β (OSTα/β), which can function as a bidirectional transport system in the basolateral membrane of the hepatocyte.^8^, ^25^ Moreover, microsomal epoxide hydrolase (mEH), a bifunctional protein that also mediated the sodium‐dependent transport of bile acids to hepatocytes,^26^ may serve as another compensatory mechanism in NTCPD affected patients.

In this study, the positive correlation between TBA levels and neonatal indirect hyperbilirubinemia, as shown in Figure S3, suggests that NTCPD is a new genetic factor related to neonatal indirect hyperbilirubinemia. Qiu et al also reported two NTCPD patients with similar presentations.^10^ Neonates have very rich bilirubin sources but immature liver function to uptake, conjugate and excrete bilirubin.^27^ On this basis, since the main function of OATP1B1/B3 is to uptake plasma bilirubin (including DBIL and IBIL) besides bile acids,^28^ hypercholanemia in NTCPD competitively inhibited the transport of bilirubin by OATPs and hence gave rise to neonatal indirect hyperbilirubinemia.^10^ This possible mechanism was further supported by the rapidly declined neonatal IBIL levels without rebounding over time in the paediatric NTCPD patients who had more mature liver function and fewer bilirubin sources.

Moreover, transient cholestatic hepatitis was found in this study to be an additional clinical presentation for NTCPD during early infancy, and this concept was also supported by the hepatohistological findings in NTCPD patients described very recently.^13^ In fact, OATP1B1/B3 had the main function of transporting DBIL,^28^ and hypercholanemia reportedly resulted in reduced expression of OATPs in the basolateral membrane of the hepatocyte in mice,^5^ hence impairing the hepatocytic uptake of DBIL from plasma and giving rise to direct hyperbilirubinemia (cholestasis). In addition, the expression of the genes *Abcc3* and *Abcc4* was significantly increased in a mice NTCPD model,^29^ and the two genes encoded MRP3 and MRP4, respectively, in the basolateral membrane of the hepatocyte in humans, functioning to pump bile acids into systemic circulation.^30^ This evidence in part explained the transiently raised DBIL and TBA levels in the cholestatic infants with NTCPD. As observed, Vit D deficiency arose as a complication of the transient cholestatic hepatitis, suggesting a transiently impaired hepatic excretion of bile acids in NTCPD infants.

Of note, all the 38 adult patients (Table S1) were symptom‐ and sign‐negative, and all the 113 paediatric NTCPD patients themselves displayed favourable clinical outcomes in this study, suggesting NTCPD is a generally benign condition. However, considering the invasive exploratory operations and expensive genetic investigations as illustrated in Table S5, the avoidance of over investigation and intervention may be important in the management of cholestatic infants with NTCPD. To address this issue, a clear understanding of its laboratory and clinical features is an essential prerequisite.

In conclusion, the findings in this study enriched the *SLC10A1* mutation spectrum and provided comprehensive insights into the phenotypic characteristics of NTCPD. With hypercholanemia as the most common clinical feature, NTCPD exhibited other age‐dependent phenotypic presentations in humans, including transient cholestatic hepatitis in early infancy and indirect hyperbilirubinemia in neonates. NTCPD should be considered and the *SLC10A1* gene should be analysed in patients with these presentations, and over investigation and intervention should be avoided in the management of NTCPD patients.

## CONFLICT OF INTEREST

We declare no conflicts of interests.

## AUTHOR CONTRIBUTIONS

Yuan‐Zong Song conceptualized and designed the study, designed the data collection instruments, critically reviewed and revised the manuscript and followed up all the patients and obtained consent for publication from the guardian. Li‐Jing Deng collected data, carried out the analyses and part of the genetic analyses, drafted the initial manuscript and revised the manuscript. Rui Liu, Mei Deng, Jian‐Wu Qiu, Muhammad Rauf Yaqub, Muhammad Atif Raza, Wei‐Xia Lin, Li Guo, and Hua Li carried out the genetic analyses. Wen‐Xian Ouyang, Ying Ouyang, Yu‐Ge Huang, Yue‐Jun Huang, Xiao‐Ling Long, Xiao‐Ling Huang, Shuang‐Jie Li collected the data. All authors approved the final manuscript as submitted and agree to be accountable for all aspects of the work.

## Supporting information

Fig S1

Fig S2

Fig S3

Table S1

Table S2

Table S3

Table S4

Table S5

## Data Availability

Data sharing not applicable to this article as no datasets were generated or analysed during the current study.
