# Supplementary figures and images for: Clinical characterization of NTCP deficiency in paediatric patients : A case‐control study based on SLC10A1 genotyping analysis
Source: Liver Int. 2021 Aug 25;41(11):2720–8. doi: 10.1111/liv.15031 (PMC9291912; doi:10.1111/liv.15031)

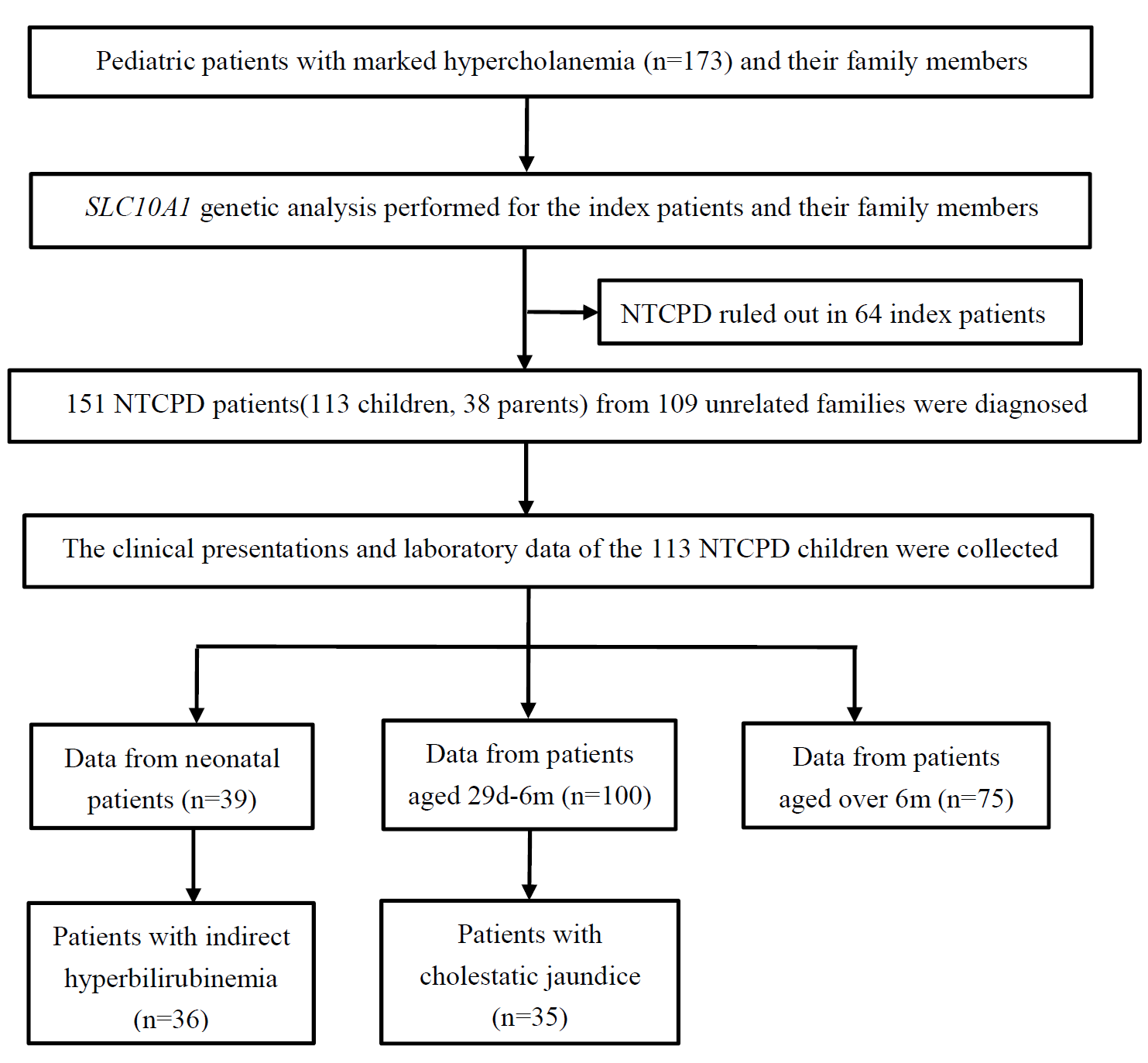

Supplement: Supplementary file 1 — Fig S1 [file LIV-41-2720-s002.tif]

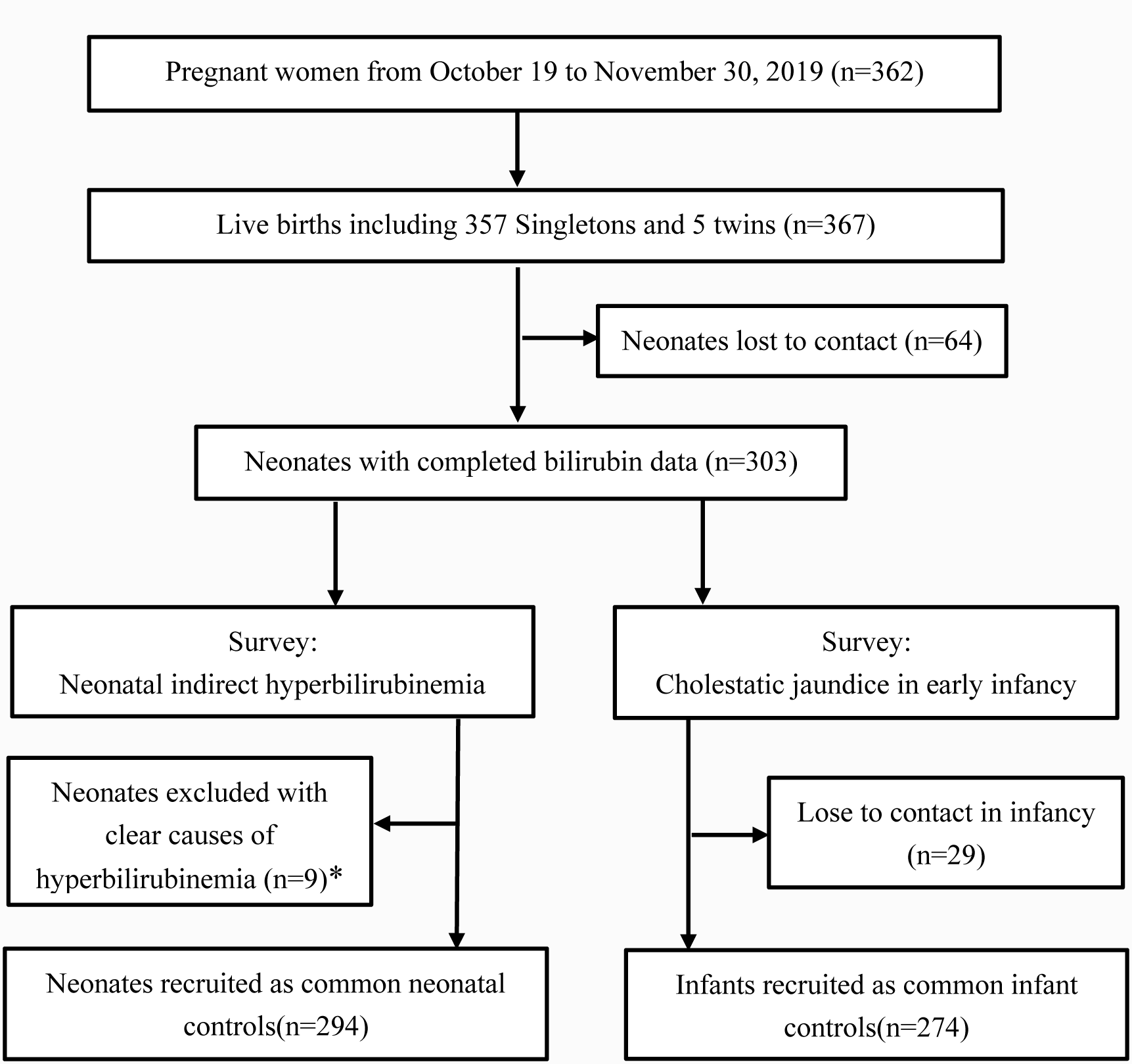

Supplement: Supplementary file 2 — Fig S2 [file LIV-41-2720-s008.tif]

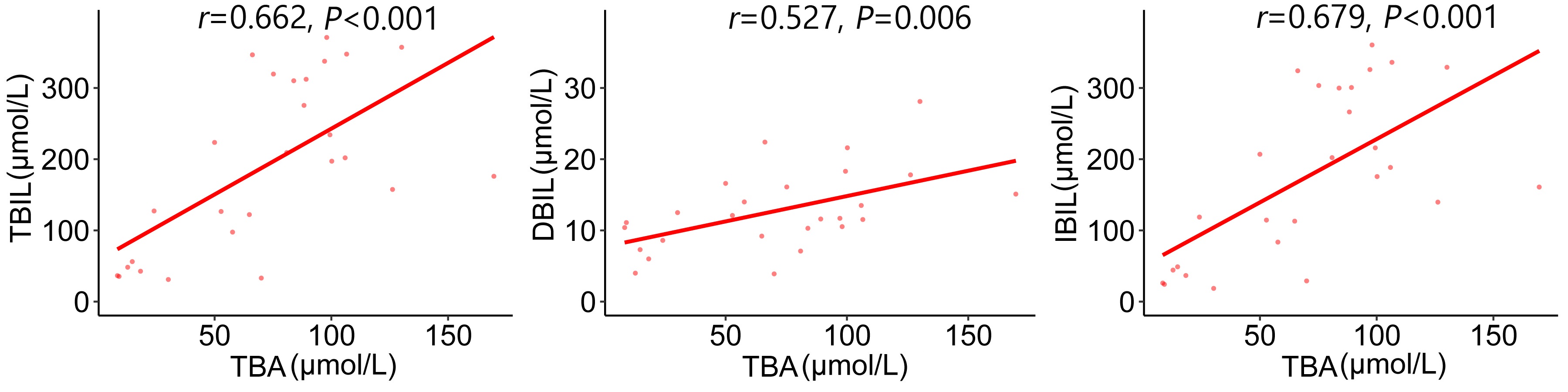

Supplement: Supplementary file 3 — Fig S3 [file LIV-41-2720-s005.jpg]
