# Supplementary material for: Clinical characterization of NTCP deficiency in paediatric patients : A case‐control study based on SLC10A1 genotyping analysis
Source: Liver Int. 2021 Aug 25;41(11):2720–8. doi: 10.1111/liv.15031 (PMC9291912; doi:10.1111/liv.15031)
Supplement: Supplementary file 4 — Table S1 [file LIV-41-2720-s006.docx]

**Supplementary Table 1**  ***SLC10A1* genotyping analysis of the 113 pediatric patients with NTCPD**

| **Family No.** | **Patient**  **No.** | **Family trios** | | |  |  | **Family No.** | **Patient**  **No.** | **Family trios** | | |
| --- | --- | --- | --- | --- | --- | --- | --- | --- | --- | --- | --- |
|  |  | **Index Patient** | **Father** | **Mother** |  |  |  |  | **Index Patient** | **Father** | **Mother** |
| 1 | N02 | c.800C>T/c.800C>T | c.800C>T/— | c.800C>T/— |  |  | 55 | N110 | c.800C>T/c.800C>T | c.800C>T/— | ***c.800C>T/c.800C>T*** |
| 2 | N04 | c.800C>T/c.800C>T | ***c.800C>T/c.800C>T*** | c.800C>T/— |  |  | 56 | N112 | c.800C>T/c.800C>T | c.800C>T/— | c.800C>T/— |
| 3 | N05 | c.800C>T/c.800C>T | c.800C>T/— | c.800C>T/— |  |  | 57 | N113 | c.800C>T/c.800C>T | c.800C>T/— | c.800C>T/— |
| 4 | N07 | c.800C>T/c.800C>T | ***c.800C>T/c.800C>T*** | ***c.800C>T/c.800C>T*** |  |  | 58 | N114 | c.800C>T/c.800C>T | c.800C>T/— | c.800C>T/— |
| 5  6 | N09  N10 | c.800C>T/c.800C>T  c.800C>T/c.800C>T | c.800C>T/—  c.800C>T/— | c.800C>T/—  ***c.800C>T/c.800C>T*** |  |  | 59 | N114B  N115 | c.800C>T/c.800C>T  c.800C>T/c.800C>T | c.800C>T/— | c.800C>T/— |
| 7 | N11 | c.800C>T/c.800C>T | c.800C>T/— | ***c.800C>T/c.800C>T*** |  |  | 60 | N118 | c.800C>T/c.800C>T | c.800C>T/— | c.800C>T/— |
| 8 | N13 | c.800C>T/c.263T>C | c.800C>T/— | c.263T>C/— |  |  | 61 | N119 | c.800C>T/c.800C>T | ***c.800C>T/c.800C>T*** | c.800C>T/— |
| 9 | N21 | c.800C>T/c.800C>T | ***c.800C>T/c.800C>T*** | c.800C>T/— |  |  | 62 | N120 | c.800C>T/c.800C>T | c.800C>T/—  ***c.800C>T/c.800C>T*** | c.800C>T/—  c.800C>T/— |
| 10 | N22 | c.800C>T/c.800C>T | ***c.800C>T/c.800C>T*** | c.800C>T/— |  |  | 63 | N121 | c.800C>T/c.800C>T |  |  |
| 11 | N23 | c.800C>T/c.800C>T | c.800C>T/— | c.800C>T/— |  |  | 64 | N123 | c.800C>T/c.800C>T | c.800C>T/— | c.800C>T/— |
| 12 | N24 | c.800C>T/c.595A>C | ***c.800C>T/c.595A>C*** | c.800C>T/— |  |  | 65 | N125 | c.800C>T/c.800C>T | ***c.800C>T/c.800C>T*** | c.800C>T/— |
| 13 | N28 | c.800C>T/c.800C>T | c.800C>T/— | ***c.800C>T/c.800C>T*** |  |  | 66 | N127 | c.800C>T/ c.800C>T | NA | c.800C>T/— |
| 14  15 | N30  N32 | c.800C>T/c.800C>T  c.263T>C/c.800C>T | c.800C>T/—  c.263T>C/— | c.800C>T/—  c.800C>T/— |  |  | 67  68 | N128  N129 | c.800C>T/c.800C>T  c.800C>T/c.800C>T | c.800C>T/—  ***c.800C>T/c.800C>T*** | c.800C>T/—  c.800C>T/— |
| 16 | N38 | c.800C>T/c.800C>T | c.800C>T/— | c.800C>T/— |  |  | 69 | N130 | c.800C>T/c.800C>T | c.800C>T/— | c.800C>T/— |
| 17 | N39a | c.800C>T/c.800C>T | c.800C>T/— | c.800C>T/— |  |  | 70 | N132 | c.800C>T/ c.800C>T | NA | c.800C>T/— |
|  | N39b | c.800C>T/c.800C>T |  |  |  |  | 71 | N133 | c.800C>T/c.800C>T | c.800C>T/— | c.800C>T/— |
| 18  19 | N40  N50 | c.800C>T/c.800C>T  c.800C>T/c.800C>T | NA  c.800C>T/— | c.800C>T/—  c.800C>T/— |  |  | 72  73 | N135  N137 | c.800C>T/c.800C>T  c.800C>T/c.800C>T | c.800C>T/—  c.800C>T/— | c.800C>T/—  ***c.800C>T/c.800C>T*** |
| 20 | N55 | c.800C>T/c.800C>T | ***c.800C>T/c.800C>T*** | c.800C>T/— |  |  | 74 | N138 | c.800C>T/c.800C>T | ***c.800C>T/c.800C>T*** | c.800C>T/— |
| 21 | N56 | c.800C>T/*c.595A>C* | *c.595A>C/ —* | c.800C>T/— |  |  | 75 | N139 | c.800C>T/c.800C>T | c.800C>T/— | c.800C>T/— |
| 22 | N58 | c.800C>T/c.800C>T | c.800C>T/— | c.800C>T/— |  |  | 76 | N140 | c.800C>T/c.800C>T | c.800C>T/— | c.800C>T/— |
| 23 | N59 | c.800C>T/c.800C>T | NA | c.800C>T/— |  |  | 77 | N142 | c.800C>T/c.800C>T | c.800C>T/— | c.800C>T/— |
| 24 | N60 | c.800C>T/c.800C>T | ***c.800C>T/c.800C>T***  c.800C>T/— | ***c.800C>T/c.800C>T***  c.800C>T/— |  |  | 78 | N144 | c.800C>T/c.800C>T | c.800C>T/— | c.800C>T/— |
| 25 | N61 | c.800C>T/c.800C>T |  |  |  |  | 79 | N145 | c.800C>T/c.800C>T | c.800C>T/— | c.800C>T/— |
| 26 | N63 | c.800C>T/c.800C>T | c.800C>T/— | c.800C>T/— |  |  | 80 | N146 | c.800C>T/c.800C>T | c.800C>T/— | c.800C>T/— |
|  | N63B | c.800C>T/c.800C>T |  |  |  |  | 81 | N147 | c.800C>T/c.800C>T | c.800C>T/— | c.800C>T/— |
| 27 | N66 | c.800C>T/c.800C>T | ***c.800C>T/c.800C>T*** | c.800C>T/— |  |  | 82 | N148 | c.800C>T/c.800C>T | c.800C>T/— | c.800C>T/— |
| 28 | N67 | c.800C>T/c.800C>T | c.800C>T/— | c.800C>T/— |  |  | 83 | N152 | c.800C>T/**c.374dupG** | **c.374dupG**/— | c.800C>T/— |
| 29 | N68 | c.800C>T/c.800C>T | c.800C>T/— | c.800C>T/— |  |  | 84 | N153 | c.800C>T/**c.374dupG** | ***c.800C>T/c.800C>T*** | **c.374dupG**/*—* |
| 30 | N71 | c.800C>T/c.800C>T | c.800C>T/— | c.800C>T/— |  |  | 85 | N154 | c.800C>T/c.800C>T | c.800C>T/— | c.800C>T/— |
| 31 | N72 | c.800C>T/c.800C>T | c.800C>T/— | c.800C>T/— |  |  | 86 | N155 | c.800C>T/c.800C>T | ***c.800C>T/c.800C>T*** | c.800C>T/— |
| 32 | N76 | c.800C>T/c.800C>T | c.800C>T/— | c.800C>T/— |  |  | 87 | N156 | c.800C>T/c.800C>T | ***c.800C>T/c.800C>T*** | ***c.800C>T/c.800C>T*** |
| 33 | N79 | c.800C>T/c.800C>T | c.800C>T/—  NA | c.800C>T/—  ***c.800C>T/c.800C>T*** |  |  | 88 | N157 | c.800C>T/c.800C>T | c.800C>T/— | c.800C>T/— |
| 34 | N80 | c.800C>T/c.800C>T |  |  |  |  | 89 | N158 | c.800C>T/c.800C>T | c.800C>T/— | c.800C>T/— |
| 35 | N81 | c.800C>T/c.800C>T | c.800C>T/— | c.800C>T/— |  |  | 90 | N159 | c.800C>T/c.800C>T | c.800C>T/— | ***c.800C>T/c.800C>T*** |
| 36 | N83 | c.800C>T/c.800C>T | c.800C>T/— | c.800C>T/— |  |  | 91 | N160 | c.800C>T/c.800C>T | c.800C>T/— | c.800C>T/— |
| 37 | N86 | c.800C>T/c.800C>T | NA | ***c.800C>T/c.800C>T*** |  |  | 92 | N161 | c.800C>T/c.800C>T | c.800C>T/— | c.800C>T/— |
| 38 | N88 | c.800C>T/c.800C>T | NA | c.800C>T/— |  |  | 93 | N162 | c.800C>T/c.800C>T | c.800C>T/— | c.800C>T/— |
| 39 | N89 | c.800C>T/c.800C>T | c.800C>T/— | c.800C>T/— |  |  | 94 | N164 | c.800C>T/c.800C>T | c.800C>T/— | c.800C>T/— |
| 40 | N90 | c.263T>C/c.800C>T | c.263T>C/— | c.800C>T/— |  |  | 95 | N165 | c.800C>T/c.800C>T | ***c.800C>T/c.800C>T*** | c.800C>T/— |
| 41 | N91 | c.800C>T/c.800C>T | c.800C>T/— | c.800C>T/— |  |  | 96 | N167 | c.800C>T/c.800C>T | c.800C>T/— | c.800C>T/— |
| 42 | N92 | c.800C>T/c.800C>T | ***c.263T>C****/****c.800C>T*** | c.800C>T/— |  |  | 97 | N169 | c.800C>T/c.800C>T | c.800C>T/— | c.800C>T/— |
| 43 | N93 | c.800C>T/c.800C>T | c.800C>T/— | c.800C>T/— |  |  | 98 | N170 | c.800C>T/c.800C>T | c.800C>T/— | NA |
| 44 | N94 | c.800C>T/c.800C>T | ***c.800C>T/c.800C>T*** | c.800C>T/— |  |  | 99 | N171 | c.800C>T/c.800C>T | c.800C>T/— | c.800C>T/— |
| 45 | N95 | c.800C>T/c.263T>C | c.800C>T/— | c.263T>C/— |  |  | 100 | N173 | c.800C>T/c.800C>T | c.800C>T/— | c.800C>T/— |
| 46 | N96 | c.800C>T/c.800C>T | c.800C>T/— | c.800C>T/— |  |  | 101 | N175 | c.800C>T/c.800C>T | c.800C>T/— | c.800C>T/— |
| 47 | N97 | c.800C>T/ c.263T>C | ***c.800C>T/c.800C>T*** | c.263T>C/— |  |  | 102 | N176 | c.800C>T/c.263T>C | ***c.800C>T/c.800C>T*** | c.263T>C/— |
|  | N97B | c.800C>T/ c.263T>C |  | c |  |  | 103 | N177 | c.800C>T/c.800C>T | ***c.800C>T/c.800C>T*** | c.800C>T/— |
| 48 | N98 | c.800C>T/c.800C>T | c.800C>T/— | c.800C>T/— |  |  | 104 | N178 | c.800C>T/c.800C>T | ***c.800C>T/c.800C>T*** | ***c.800C>T/c.800C>T*** |
| 49  50 | N99  N100 | c.800C>T/c.800C>T  c.800C>T/c.800C>T | c.800C>T/—  c.800C>T/— | ***c.800C>T/c.800C>T***  c.800C>T/— |  |  | 105  106 | N179  N180 | c.800C>T/c.800C>T  c.800C>T/c.800C>T | c.800C>T/—  c.800C>T/— | ***c.800C>T/c.800C>T***  c.800C>T/— |
| 51 | N102 | c.800C>T/c.800C>T | c.800C>T/— | c.800C>T/— |  |  | 107 | N186 | c.800C>T/c.800C>T | c.800C>T/— | c.800C>T/— |
| 52 | N106 | c.800C>T/c.800C>T | c.800C>T/— | c.800C>T/— |  |  | 108 | N194 | c.800C>T/**c.682_683delCT** | c.800C>T/— | **c.682_683delCT**/— |
| 53 | N107 | c.800C>T/c.800C>T | c.800C>T/— | c.800C>T/— |  |  | 109 | N195 | c.800C>T/c.263T>C | c.800C>T/— | c.263T>C/— |
| 54 | N109 | c.800C>T/c.800C>T | c.800C>T/— | ***c.800C>T/c.800C>T*** |  |  |  |  |  |  |  |

In bold and underlined were the novel *SLC10A1* variants, and in italic bold, the genotypes for the 38 adult patients with NTCPD. In family 21, N39a and N39b were twins. NA: not analyzed. All NTCPD patients were homozygous for the variant c.800C>T other than N13, N24, N32, N56, N90, N95, N97, N97B, N152, N153, N176, N194, N195 who were compound heterozygotes of c.800C>T and other *SLC10A1* variants c.263T>C, c.595A>C, c.374dupG or c.682_683delCT. Some genotypes (those for N02, N13, N32, N39a, N39b, N60, N80, N24, N56) in this table have been reported previously[9,10,14, 15, 16].
