# Supplementary material for: Clinical characterization of NTCP deficiency in paediatric patients : A case‐control study based on SLC10A1 genotyping analysis
Source: Liver Int. 2021 Aug 25;41(11):2720–8. doi: 10.1111/liv.15031 (PMC9291912; doi:10.1111/liv.15031)
Supplement: Supplementary file 5 — Table S2 [file LIV-41-2720-s004.docx]

**Supplementary Table 2** **Major presentations at first referrals**

| Initial presentations | Number of Index patients | Proportion(%) | Index patients |
| --- | --- | --- | --- |
| Jaundice | 57 | 50.44 | N02, N04, N05, N07, N09, N11, N13, N23, N28, N30, N32, N38, N39a, N39b, N40, N55, N58, N59, N61, N63, N67, N68, N71, N81, N83, N88, N89, N92, N95, N96, N98, N102, N106, N107, N109, N110, N119, N123, N125, N130, N133, N137, N139, N140, N147, N153, N157, N158, N159, N161, N165, N173, N176, N177, N178, N179, N186 |
| Infections(Pneumonia, Influenza, Enteritis, lymphadenitis, et al) | 17 | 15.04 | N50, N72, N91, N93, N97, N100, N114, N127, N128, N132, N152, N155, N160, N162, N171, N175, N194 |
| Premature infants | 14 | 12.39 | N56, N80, N86, N94, N112, N113, N115, N118, N120, N129, N138, N156, N170, N195 |
| High-risk infants | 8 | 7.08 | N10, N21, N60, N90, N99, N121, N142, N144 |
| Intrauterine growth retardation | 2 | 1.77 | N135, N156 |
| Abnormal liver function on health examination | 7 | 6.19 | N24, N66, N79, N145, N154, N164, N167 |
| Gastrointestinal presentations (abdominal distention, vomiting, anal atresia) | 5 | 4.43 | N22, N76, N146, N148, N180 |
| With siblings previously diagnosed with NTCPD | 3 | 2.66 | N63B, N97B, N114B |
| Total | 113 | 100% |  |

.
