# Supplementary material for: Clinical characterization of NTCP deficiency in paediatric patients : A case‐control study based on SLC10A1 genotyping analysis
Source: Liver Int. 2021 Aug 25;41(11):2720–8. doi: 10.1111/liv.15031 (PMC9291912; doi:10.1111/liv.15031)
Supplement: Supplementary file 6 — Table S3 [file LIV-41-2720-s003.docx]

**Supplementary Table 3 ACMG Classification of the *SLC10A1* variants detected in this study**

| **No.** | **Variants** | **First case** | **Classification** | **ACMG criteria** |
| --- | --- | --- | --- | --- |
| 1 | c.800C>T(p.Ser267Phe) | Deng M, et al. [9]. | Pathogenic | **2PS** PS1: Well-established in vitro functional studies [*]; PS2: Significantly increased prevalence in the patients in this study [94.50%(206/218)] than in controls [4.7%(7/150) in Ref. 9, χ^2^=290.44, *P<0.001*].  **1PM** PM3: Detected in trans with a pathogenic variant[11].  **2PP** PP3: Multiple lines of computational evidence support a deleterious effect[9]; PP4: Patient’s phenotype or family history is highly specific for this disease[9]. |
| 2 | c.263T>C(p.Ile88Thr) | Qiu JW, et al.  [10]. | Pathogenic | **1PS** Well-established in vitro functional studies [**].  **2PM** PM2: At extremely low frequency in relevant databases[10];PM3: Detected in trans with a pathogenic variant[10].  **3PP** PP1:Cosegregation with disease in multiple affected family members[10];PP3: Multiple lines of computational evidence support a deleterious effect[10];PP4: Patient’s phenotype or family history is highly specific for this disease[10]. |
| 3 | c.595A>C(p.Ser199Arg) | Li H, et al.  [16]. | Likely Pathogenic | **2PM** PM2: At extremely low frequency[16];PM3: detected in trans with a pathogenic variant [16].  **3PP** PP1:Cosegregation with disease in multiple affected family members[16];PP3: Multiple lines of computational evidence support a deleterious effect[16];PP4: Patient’s phenotype or family history is highly specific for this disease[16]. |
| 4 | c.374dupG  (p.Cys125TrpfsTer23) | This study | Pathogenic | **1PVS** Null variant.  **2PM** PM2: Absent from controls;PM3: Detected in trans with a pathogenic variant.  **1PP** PP4: Patient’s phenotype or family history is highly specific for a disease with a single genetic etiology. |
| 5 | c.682_683delCT  (p.Leu228AspfsTer49) | This study | Pathogenic | **1PVS** Null variant.  **2PM** PM2:Absent from controls;PM3: Detected in trans with a pathogenic variant.  **1PP** PP4: Patient’s phenotype or family history is highly specific for a disease with a single genetic etiology. |

* Ho RH, Leake BF, Roberts RL, Lee W, and Kim RB. Ethnicity-dependent polymorphism in Na+-taurocholate cotransporting polypeptide (SLC10A1) reveals a domain critical for bile acid substrate recognition. J Biol Chem. 2004;279:7213-22.

** Russell LE, Zhou Y, Lauschke VM, and Kim RB. In Vitro Functional Characterization and in Silico Prediction of Rare Genetic Variation in the Bile Acid and Drug Transporter, Na(+)-Taurocholate Cotransporting Polypeptide (NTCP, SLC10A1). Mol Pharm. 2020;17:1170-81.
