# Supplementary material for: Clinical characterization of NTCP deficiency in paediatric patients : A case‐control study based on SLC10A1 genotyping analysis
Source: Liver Int. 2021 Aug 25;41(11):2720–8. doi: 10.1111/liv.15031 (PMC9291912; doi:10.1111/liv.15031)
Supplement: Supplementary file 7 — Table S4 [file LIV-41-2720-s007.docx]

**Supplementary Table 4 Comparison of serum biochemical indices between patients and controls**

| **Indices** | **Reference range** | **Pediatric patients** | **Healthy children** | **Z** | ***p*** |
| --- | --- | --- | --- | --- | --- |
| Number | - | 113 | 52 | - | - |
| M:F^#^ | - | 70:43 | 24:28 | 3.01 | 0.083 |
| Ages* | - | 0.3(0, 70), n=112 | 12(1, 180), n=52 | -8.35 | <0.001 |
| TBA | 0-10 μmol/L | 124.9(2.9, 737.8), n=865 | 5.04±3.03, n=47 | -11.50 | <0.001 |
| CG | 0.4-2.78mg/L | 84.8(0.5, 353), n=234 | 2.45(0.2, 7.7),n=38 | -9.71 | <0.001 |
| TBIL | 5.1-23μmol/L | 14.5(1.8, 540.8), n=768 | 8.8(2.5, 28.2), n=47 | -3.91 | <0.001 |
| DBIL | 0.6-6.8 μmol/L | 5.3(0, 394.1), n=838 | 2.6(0.8, 8.8), n=47 | -4.87 | <0.001 |
| IBIL | 1.7-17 μmol/L | 8.6(0.5, 447.5), n=729 | 6.1(0.6, 22.7), n=47 | -2.84 | 0.004 |
| ALT | 5-40U/L | 27.4(3, 1161), n=737 | 21.2±8.4, n=47 | -4.14 | <0.001 |
| AST | 5-50U/L | 43(12, 1295), n=705 | 33.4±8.6,n=47 | -5.51 | <0.001 |
| GGT | 10-60U/L | 36.5(3, 753), n=692 | 12(6, 290), n=46 | -4.92 | <0.001 |
| Vit D | >10 ng/mL | 28.0(4, 83.9), n=212 | 33.7±10.3 , n=36 | -2.71 | 0.007 |
| ALP | 150-700U/L | 274(56, 1939), n=680 | 288.5(154, 652), n=48 | -1.00 | 0.319 |
| TP | 65.0-85.0 g/L | 59.2±6.9, n=662 | 64.0±8.1, n=46 | -3.8 | <0.001 |
| ALB | 40.0-55.0 g/L | 41.5(10.6, 53.7), n=706 | 42.7±3.5, n=46 | -2.51 | 0.012 |
| GLB | 20.0-40.0 g/L | 17.3(4.97, 42.7), n=619 | 21.2±5.5, n=46 | -3.97 | <0.001 |
| ADA | 4-24U/L | 17.9±8.1, n=358 | 17.5(6, 30), n=46 | -1.01 | 0.312 |
| TG | 0.56-1.70mmol/L | 1.2(0.11, 8.3), n=205 | 1.1±0.5, n=18 | -1.427 | 0.154 |
| Tchol | 3.1-5.70mmol/L | 3.6(1.1, 8.2), n=217 | 4.0±0.7, n=18 | -1.476 | 0.140 |
| HDL | 0.91-2.05mmol/L | 1.1(0.3, 2.6) , n=202 | 1.2±0.3, n=18 | -1.583 | 0.114 |
| LDL | 1.57-3.76mmol/L | 2.0(0.3, 6.1), n=201 | 2.2±0.5, n=18 | -0.674 | 0.501 |
| APOA | 1-1.6g/L | 1.2±0.4, n=167 | 1.4±0.3, n=17 | -2.283 | 0.022 |
| APOB | 0.6-1.08g/L | 0.7(0.1, 2.6), n=167 | 0.8±0.2, n=17 | -0.760 | 0.447 |
| APOE | 27-49mg/L | 55.5(20.6, 370.6), n=108 | 51±22.1, n=13 | -1.502 | 0.133 |
| SOD | 129-216U/mL | 201(131, 1170), n=289 | 183.5±25.1, n=45 | -3.829 | <0.001 |
| FN | 180-280mg/L | 181.5±39.6, n=231 | 190.5±32.5, n=42 | -1.617 | 0.106 |
| RBP | 25-70mg/L | 23.4(0.02, 80.2), n=265 | 26.6±7.8, n=43 | -1.366 | 0.172 |
| CHE | 4000-12900U/L | 8340±2289, n=422 | 8650(5991,15774), n=46 | -1.195 | 0.232 |
| CER | 0.2-0.6g/L | 0.3±0.1, n=248 | 0.3(0.2, 0.6), n=42 | -0.700 | 0.484 |
| Zinc | 6.5-25.5μmol/L | 8.8±2.9, n=239 | 8.4(3, 18.3), n=45 | -0.424 | 0.671 |

In this table, ^#^Comparison of the male/female ratios was made by using Chi-square test; * The ages in children were presented in months when the first biochemistry analysis was performed. M:F denoted Males: Females.
