# Supplementary material for: Clinical characterization of NTCP deficiency in paediatric patients : A case‐control study based on SLC10A1 genotyping analysis
Source: Liver Int. 2021 Aug 25;41(11):2720–8. doi: 10.1111/liv.15031 (PMC9291912; doi:10.1111/liv.15031)
Supplement: Supplementary file 8 — Table S5 [file LIV-41-2720-s001.docx]

**Supplementary Table 5 Detailed information for the patients with special examination**

| Patients | Ages* | Gender | [Special](javascript:;) [Examination](javascript:;) | TBA(μmol/L) |
| --- | --- | --- | --- | --- |
| N02 | 31m | Male | NGS | 405.8 |
| N05 | 2.5m | Male | Laparoscopic biliary exploration  Liver biopsy, Cholangiography, NGS | 106.02 |
| N28 | 1.7m | Male | Exploratory laparotomy  Cholecystostomy, Cholangiography | 85.1 |
| N76 | 10.5m | Male | Liver puncture | 165.7 |
| N96 | 1.5m | Male | Laparoscopic Biliary exploration, Liver biopsy | 180.19 |
| N127 | 11m | Male | NGS | 406.4 |
| N128 | 7.4m | Male | NGS | 255 |
| N133 | 2.5m | Male | NGS, Liver puncture | 141.6 |
| N152 | 70m | Male | NGS | 160.8 |
| N153 | 5m | Male | NGS | 265.2 |
| N178 | 1.5m | Female | NGS | 148.3 |

* When the special examination was performed; m: month; NGS: next generation sequencing.
